# Prolonged infection triggered by dormant *Mycobacterium tuberculosis*: Immune and inflammatory responses in lungs of genetically susceptible and resistant mice

**DOI:** 10.1371/journal.pone.0239668

**Published:** 2020-09-24

**Authors:** Tatiana Kondratieva, Margarita Shleeva, Marina Kapina, Elvira Rubakova, Irina Linge, Alexander Dyatlov, Elena Kondratieva, Arseny Kaprelyants, Alexander Apt

**Affiliations:** 1 Laboratory for Immunogenetics, Central Institute for Tuberculosis, Moscow, Russia; 2 Federal Research Centre "Fundamentals of Biotechnology", Russian Academy of Sciences, A. N. Bach Institute of Biochemistry, Moscow, Russia; Rutgers Biomedical and Health Sciences, UNITED STATES

## Abstract

We developed an approach for substantial attenuation of *Mycobacterium tuberculosis* by prolonged culturing under gradually acidifying conditions. Bacteria subjected to acidification lost the capacity to form colonies on solid media, but readily resuscitated their growth in the murine host, providing a useful model to study *in vivo* development of infection mimicking latent and reactivation tuberculosis (TB) in humans. Here we characterize biomarkers of lung pathology and immune responses triggered by such attenuated bacteria in genetically TB-susceptible and resistant mice. In susceptible I/St mice, CFU counts in lungs and spleens were ~1.5-log higher than in resistant B6 mice, accompanied by diffuse pneumonia and excessive lung infiltration with highly activated CD44^+^CD62L^-^ T-lymphocytes resulting in death between months 7–9 post challenge. B6 mice were characterized by development of local inflammatory foci, higher production of pro-inflammatory IL-6 and IL-11 cytokines and a more balanced T-cell activation in their lungs. CFU counts remained stable in B6 mice during the whole 18-mo observation period, and all mice survived. Thus, we established a mouse model of fatal reactivation TB *vs*. indefinite mycobacterial possession after identical challenge and characterized the features of immune responses in the lung tissue underlining these polar phenotypes.

## 1. Introduction

It is generally accepted that among immune sufficient humans not more than 3–10 per cent of individuals infected with *M*. *tuberculosis* eventually develop clinical disease [1]. Since the cases of complete spontaneous mycobacterial eradication are rare [2], about 90 per cent of infected individuals without clinical manifestations comprise a reservoir of latent tuberculosis infection (LTBI). The size of this reservoir is enormous: it was estimated that approximately 25% of global population is latently infected with *M*. *tuberculosis* [3]. In some of these individuals, tuberculosis (TB) infection transits to the active state, becomes contagious and seriously affects epidemiological situation [4]. Thus, the problem of LTBI identification, treatment and prevention is of utmost importance, especially since the latent, metabolically passive state is thought to be untreatable with currently existing TB drugs. Meanwhile, too little is known about the mechanisms of protective immunity to and pathogenesis of TB in general and LTBI in particular. Success in identification of essential immune and inflammatory mechanisms should help to dissect pathogenic biochemical pathways and assess the performance of novel vaccines and drugs using reliable biological correlates [5]. Comparison of human data with observations obtained in animal model systems will certainly facilitate our understanding of LTBI biology [6]; however, modelling LTBI appeared to be a complicated task.

For decades, several *in vitro* approaches have been applied to study stable mycobacterial persistence under pressure of different stresses, aiming to mimic survival within host granulomata *in vivo*, where *M*. *tuberculosis* is exposed to stressful anaerobic and nutrient deprived conditions (reviewed in [7]). However, neither *in vitro* model mimics these conditions accurately. This is not surprising, given the lack of immune host cells, proper 3D structure and the balance between intracellular and extracellular bacterial populations in corresponding experimental settings. Development of adequate *in vitro* models of the lung granuloma ([8, 9], reviewed in [10]), is still in its infancy and has not resulted in breakthrough in our knowledge yet. Two most common *in vivo* experimental approaches comprise reactivation disease after antibiotic treatment withdrawal (Cornell model) in a few mouse strains [11–14], and chronic disease after a low-dose aerosol challenge in B6 mice [15]. The Cornell-like models are applicable to the problem of TB reactivation in patients after “successful” chemotherapy rather than to the *bona fide* LTBI. However, by using TB-susceptible I/St mouse strain and an intensive treatment regimen, we obtained a mouse phenotype closely resembling LTBI: resolution of lung pathology without total eradication of mycobacteria and their DNA from the lung tissue. Remarkably, identical manipulations in TB-resistant B6 mice resulted in their complete recovery, with no signs of residual infection [13, 14]. These observations indicate that successful modelling of TB diversity in mice–necrotic, chronic, non-progressive and other forms of infection–requires a thorough selection of mouse strains [16, 17].

Another factor affecting the accuracy of experimental models is the duration of experiments. In humans, TB infection in most cases progresses slowly, and one of disadvantages of widely applied TB models is a relatively short duration of the disease, which kills mice and guinea pigs within 5–9 mo after aerosol challenge with the most commonly used 50–100 CFU inoculum [18]. The infective dose of *M*. *tuberculosis* in humans was estimated as 1–5 bacilli [19], so it is quite expected that the LTBI-like state does not develop in animals challenged with approximately 1.5-log higher doses. Although it was firmly established that inhalation of as few as two *M*. *tuberculosis* CFU is sufficient to initiate infection in B6 inbred mice [20], this, potentially very useful, refined approach is not used for LTBI modelling, presumably because only about one third of exposed mice get the infection, making experiments extremely bulky, laborious and time consuming.

Assuming that as longer is the duration of experimental infection, the higher is precision of its dynamic assessment and relevance to the LTBI problem, we began to use *M*. *tuberculosis* strains with substantially diminished virulence, which induce very slowly developing disease in mice. Two main approaches of *M*. *tuberculosis* H37Rv attenuation were applied: genetic attenuation caused by knocking out genes of the *Rpf* family involved in regulation of mycobacterial growth [21–23], and a prolonged exposure to a gradual acidification *in vitro*, resulting in a so-called “non-culturable”, dormant-like state of mycobacteria [24, 25]. Using the first approach, we revealed previously unknown features of early and late lung inflammation that differentiate genetically susceptible and resistant mice. In addition, we observed gradually declining lung CFU curve and zero mortality in resistant mice [23]. Attenuation by acidification was only superficially characterized; however, substantial differences in CFU dynamics between susceptible and resistant mice were evident [26]. Meanwhile, being technically simple, *in vitro* attenuation looks like an attractive option for further work since it could be easily performed in different laboratories with any mycobacterial strain or species. This resolves a complicated problem of transportation of genetically manipulated virulent strains between research teams. In addition, we recently characterized the proteome of *in vitro* attenuated *M*. *tuberculosis*, which provides the grounds for further dissection of physiological changes that occur during mycobacterial acidification [27].

Here, we present and discuss biomarkers of immune and inflammatory responses in the lungs of genetically susceptible and resistant mice infected with “non-culturable” *M*. *tuberculosis* H37Rv subjected to prolonged incubation in gradually acidifying cultural conditions.

## 2. Materials and methods

**Mice** of inbred strains I/StSnEgYCit (I/St) and C57BL/6JCit (B6) were bred and maintained under conventional conditions at the Animal Facilities of the Central Institute for Tuberculosis (Moscow, Russia) in accordance with guidelines from the Russian Ministry of Health # 755, and under the NIH Office of Laboratory Animal Welfare (OLAW) Assurance #A5502-11. Water and food were provided *ad libitum*. Female mice of 10–12 week of age in the beginning of experiments were used. All experimental procedures were approved by the Central Institute for Tuberculosis Animal Care committee (IACUC protocols #2, 7, 8, 11, approved on February 7, 2018).

### 2.1. Mycobacteria and infection

Development of *M*. *tuberculosis* strain attenuated by a prolonged exposure to gradually acidifying cultural conditions (hereafter–*Mtb*-acid) was described earlier in detail [24, 26]. Briefly, *M*. *tuberculosis* strain H37Rv was initially grown for 8 days in the Middlebrook 7H9 liquid medium supplemented with 0.05% Tween 80 and 10% ADC (albumin, dextrose, catalase) (all components from Himedia, India). One milliliter of this stock was added to 200 ml of modified Sauton’s medium containing (per liter): KH_2_PO_4_−0.5 g; MgSO_4_ x 7H_2_O - 1.4 g; L-asparagine—4 g; glycerol—2 ml; ferric ammonium citrate—0.05 g; citric acid—2 g; 0,2% glycerol, 1% ZnSO_4_ x 7H_2_O - 0.1 ml. The medium was supplemented with 0.5% BSA, 0.025% tyloxapol and 5% glucose, and adjusted to pH = 6.0–6.2 using 1M NaOH. 200ml of this culture were incubated with shaking at 200 rpm in a 500 ml flask at 37°C for 30–50 days, with regular pH assessments. After cultural pH reached 6.0–6.2 (30–45-d incubation), 2-(N-morpholino)-ethanesulfonic acid (MES) was added at the final concentration of 100mM to prevent further acidification during storage. Fifty-ml samples were transferred to 50-ml plastic tubes, tightly-capped and kept under static conditions without agitation at the room temperature for 5mo (before challenge of mice) and 13mo (before confirmation of resuscitation capacity in the presence of supernatants from the log-phase mycobacterial cultures). Results demonstrating “non-culture-ability” of mycobacteria established according this protocol and conditions of their growth resuscitation were described elsewhere [26].

Mice were infected intravenously with ~10^6^ bacteria per mouse in 200μl of sterile PBS. Since the numbers of dormant mycobacteria cannot be assessed by CFU counts, the infecting dose was estimated by direct microscopic evaluation using a cell-counting chamber, as previously described [28]. This method provides minimal deviations between individual probes.

### 2.2. CFU counts, survival time and lung pathology

At indicated time points following infection, spleens and identical lobes of the right lungs from individual mice were homogenized in 2.0 ml of sterile saline, and 10-fold serial dilutions of 0.2 ml samples were plated on Dubos agar (Difco) and incubated at 37°C for 20–22 days before CFU were counted. Survival time was monitored daily starting month 3 post-infection. Left lungs were frozen at the regimen of –60°C to –20°C temperature gradient in the electronic Cryotome® (ThermoShandon, UK), and serial 6–8μm-thick sections were made across the widest area. Sections were fixed with ice-cold acetone and stained with hematoxylin-eosin. Slides were examined by the experienced pathologist (EK) and photographed using Axioskop40 microscope and AxioCamMRc5 camera (Carl Zeiss, Berlin, Germany).

### 2.3. Lung cell suspensions

Lungs were enzymatically digested as described previously [29]. Briefly, blood vessels were washed out with 0.02% EDTA-PBS by the heart perfusion via cut *vena cava*, lungs removed, sliced into 1–2 mm^3^ pieces and incubated at 37°C for 90 min in supplemented RPMI-1640 containing 200 U/ml collagenase and 50 U/ml DNase-I (Sigma, MO). Single cell suspensions from four mice were obtained individually, washed twice in HBSS containing 2% FCS and antibiotics. 3 x 10^5^ cells were used for the assessment of surface phenotypes and the remaining sample was used for cell culturing.

### 2.4. Cytokine analyses

2 x 10^6^/ml lung cells were cultured in wells of 24-well plates for 48 hours in the presence of 10 μg/ml mycobacterial ultrasonic disintegrate established as previously described [30]. Cytokine contents in supernatants were assessed in the ELISA format using ELISA MAX kits (Biolegend, Germany) for IL-6, IL-10, IFN-γ, TNF-α and DuoSetELISA kit (R&D systems, USA) for IL-11 according to the manufacturers' instructions.

**Cell phenotypes** were assessed using FACS-Callibur flow cytometer (BD) and the following labeled antibodies in different combinations:

1. From BD-Pharmingen, San-Jose, CA: anti-CD3/FITC-hamster anti-mouse CD3 (clone 145-2C11), PerCp-anti-CD8 (clone 53–6.7), PE-anti-CD19 (clone1D3), PE-anti-CD4 (clone L3T4), APC-anti-CD62L (clone MEL-14);
2. From Biolegend, San Diego, CA: PerCp-anti-CD4 (clone GK 1.5), FITC-anti-CD44 (clone IM7), FITC-antiLy-6G (clone 1A8), Alexa-anti-CD8 (clone53-6.7), PE-anti-F4/80 (clone BM8);

**Statistics** was assessed by the GraphPad Prism 7.0 program, applying the ANOVA test, with further validation by Tukey's range test. *P*< 0.05 was considered statistically significant.

## 3. Results

### 3.1. Mycobacterial growth and lung inflammation

In our previous study, we assessed the persistence of similarly attenuated mycobacteria in organs of susceptible and resistant mice at month 8 post challenge and obtained mortality curves for susceptible mice which all succumbed to infection by month 10 [26]. In the present work, we evaluated how parameters of infection differ between mouse strains at month 3 post challenge (when all mice are visibly healthy) and what are the features of pathology and immune responses triggered by the *Mtb*-acid strain in the lungs at month 7 (when month of the two strains display visibly different symptoms of infection). In addition, we assessed whether or not dormant mycobacteria are able to kill genetically resistant mice and what are the features of lung inflammation in these mice at the very late phase of infection (18mo).

**Fig 1:**
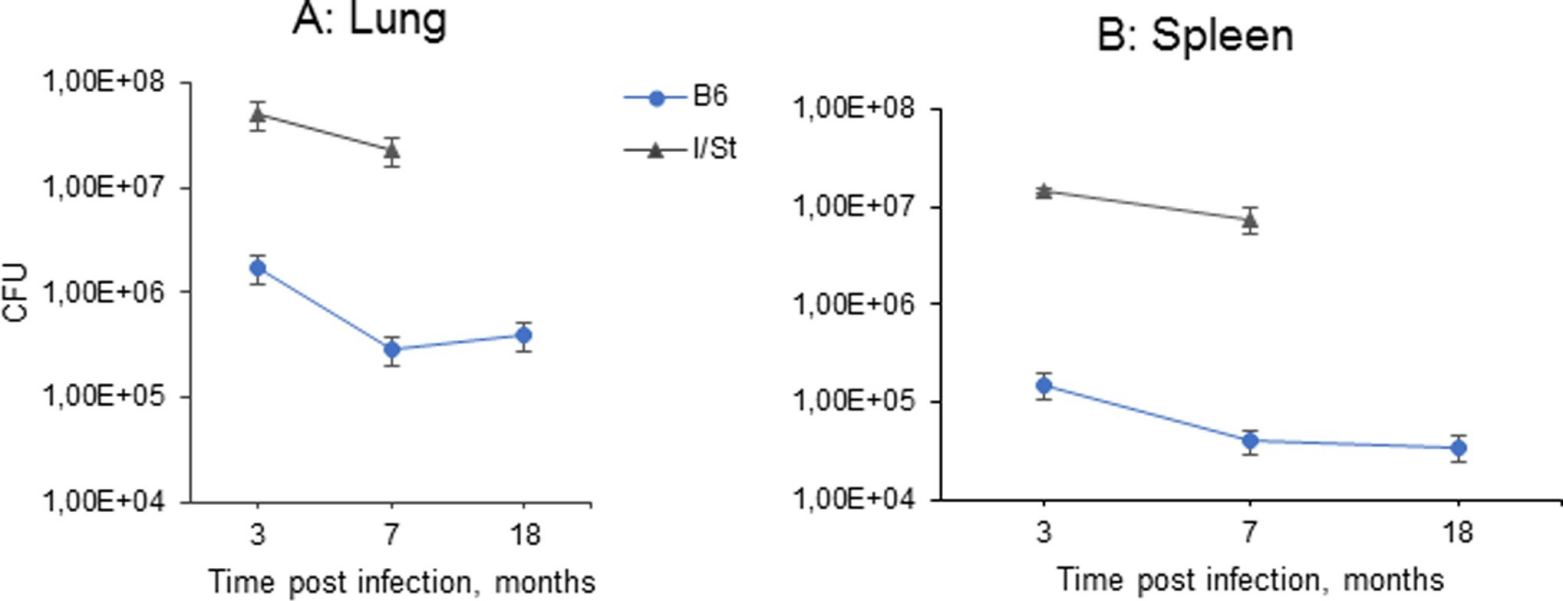
Mycobacterial persistence in lungs (A) and spleens (B) of I/St and B6 mice infected with *Mtb*-acid strain. Results for five mice per group per time point (four I/St mice per the 7-mo point) are presented as mean ± SD, inter-strain differences were highly significant (*P* < 0.0001, ANOVA).

As shown in Fig 1, 3mo post challenge, mycobacterial CFU counts in I/St and B6 mice differed ~1.5-log for lungs and ~2.0-log for spleens (*P* < 0.0001, ANOVA). The absolute numbers of CFU in organs at this time point were similar to those observed in B6 and I/St mice challenged via respiratory tract with a low dose of fully virulent *M*. *tuberculosis* [13]. This result suggests that mycobacteria attenuated *in vivo* rapidly restored their multiplication and CFU formation capacities even in genetically resistant mice, although the numbers of bacilli that survived early responses of the host were strikingly different in resistant and susceptible animals.

**Fig 2:**
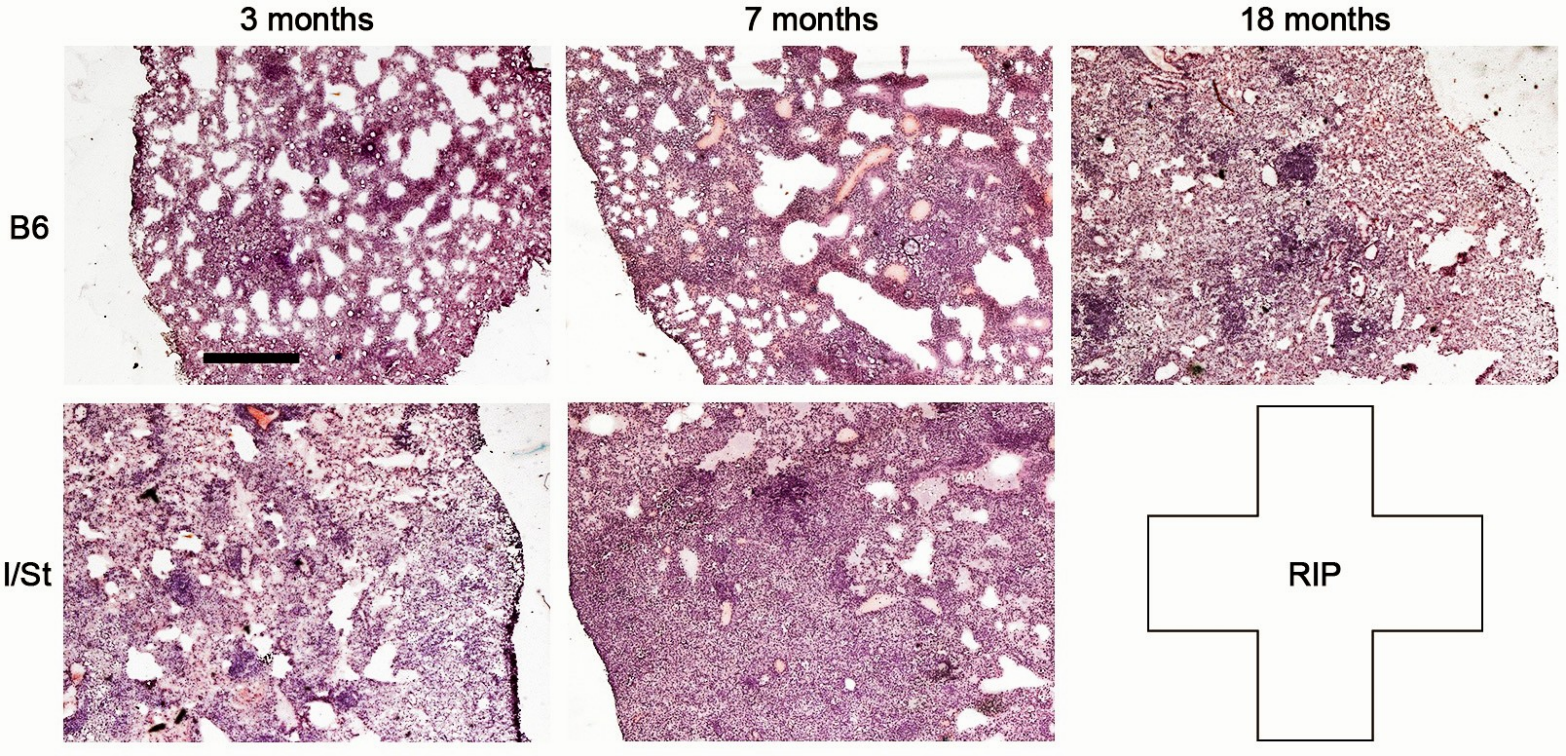
Dynamics of lung pathology in I/St and B6 mice infected with *Mtb*-acid strain. Lung tissue cryosections from 3 mice per group per time point were prepared as described in Material and Methods, representative slides are displayed at the low magnification (X 37.5) to provide synoptic pictures (~25% of the whole organ). Scale bar = 1000μm.

The differences in lung CFU counts corresponded to different dynamics of lung pathology. As shown in Fig 2, at 3mo post challenge alterations in lung tissue architecture in B6 mice were mild and appeared exclusively as small aggregates of infiltrating cells and moderate thickening of alveolar septa. In I/St mice, numerous foci of cell infiltration started to fuse forming the picture of developing pneumonia. By month 7, striking differences between mice of the two strains were evident: B6 mice were still able to control lung pathology and retain major areas of breathing tissue, whereas large zones of diffuse pneumonia combined with poorly delineated cellular aggregates were seen in lungs of I/St mice. Very soon, I/St mice started succumb to infection (mean survival time = 256 ± 37 days). B6 mice survived up to the end of experiment (no deaths or significant body weight loss at 18mo post challenge), and their lung pathology was similar to that displayed by I/St mice at 3mo.

### 3.2. Immune cells in the lung tissue

**Fig 3:**
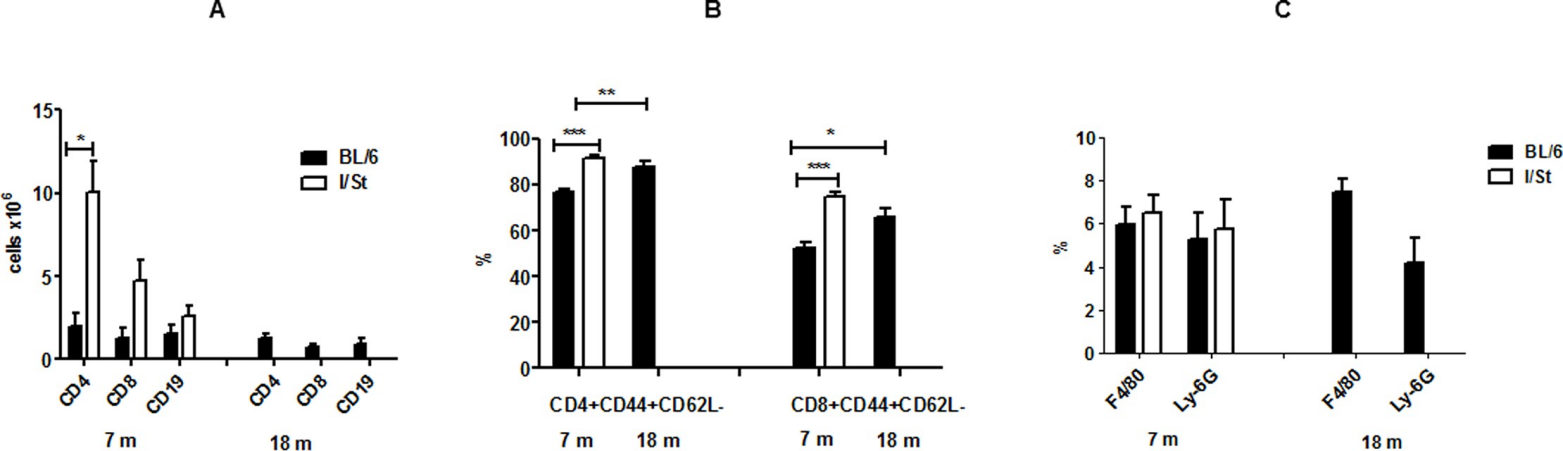
Infiltration of lungs with immune cells. The content of lymphocytes (A), phagocytes (B), and activated T-lymphocytes (C) in the lungs from five B6 at months 7 and 18 and four I/St mice at month 7 (mortality has already started in this group) mice was assessed individually by flow cytometry. Results are displayed as: the number of positive cells ± SD in the population gated for lymphocytes (A); as the per cent of macrophages and neutrophils in the total lung cell population (B); as the per cent of activated CD44^+^CD62L^-^ cells in the population of gated CD3^+^ T lymphocytes (C). Asterisks correspond to *P*: * = <0.05, ** = <0.01, *** = <0.001, ANOVA.

Since the picture of lung pathology strikingly differed in mice of the two strains at month 7 post infection, we quantitatively compared cellular responses in the lung tissue. We first assessed the sizes of main immune cell populations by flow cytometry. As shown in Fig 3A, significantly (*P* = 0.02, ANOVA) more CD4^+^ T lymphocytes infiltrated lungs of I/St compared to B6 mice. Suggestive difference (*P* = 0.08, ANOVA) was observed for CD8^+^ T cells, whereas infiltration with B cells (Fig 3A), macrophages and neutrophils (Fig 3B) was similar in the two strains. T cell populations were not only more bulky in I/St lungs, but the proportion of activated CD44^+^CD62L^-^ T cells was significantly higher in I/St mice (*P* < 0.001 for CD4^+^ and < 0.03 for CD8^+^ population, ANOVA); for CD4^+^ lung T cell, population activated cells comprised ~92% of their total amount (Fig 3C). This corresponds well to stronger and less focused lung tissue inflammation and T-cell stimulation due to a higher mycobacterial burden in I/St mice.

Evaluation of the lung immune cell content in B6 mice at 18mo post challenge demonstrated that the population sizes of all lymphocytes and phagocytes remained stable (Fig 3), despite notable enlargement of inflammatory foci between month 7 and 18 (Fig 2). This suggests that at the late phase of well-controlled infection immune cells are concentrated at restricted areas of the lung tissue: within tuberculous foci or in their vicinity. In line with this conclusion, the proportion of activated CD4^+^ and CD8^+^ T lymphocytes significantly increased between these time points (Fig 3).

### 3.3. Cytokine production

**Fig 4:**
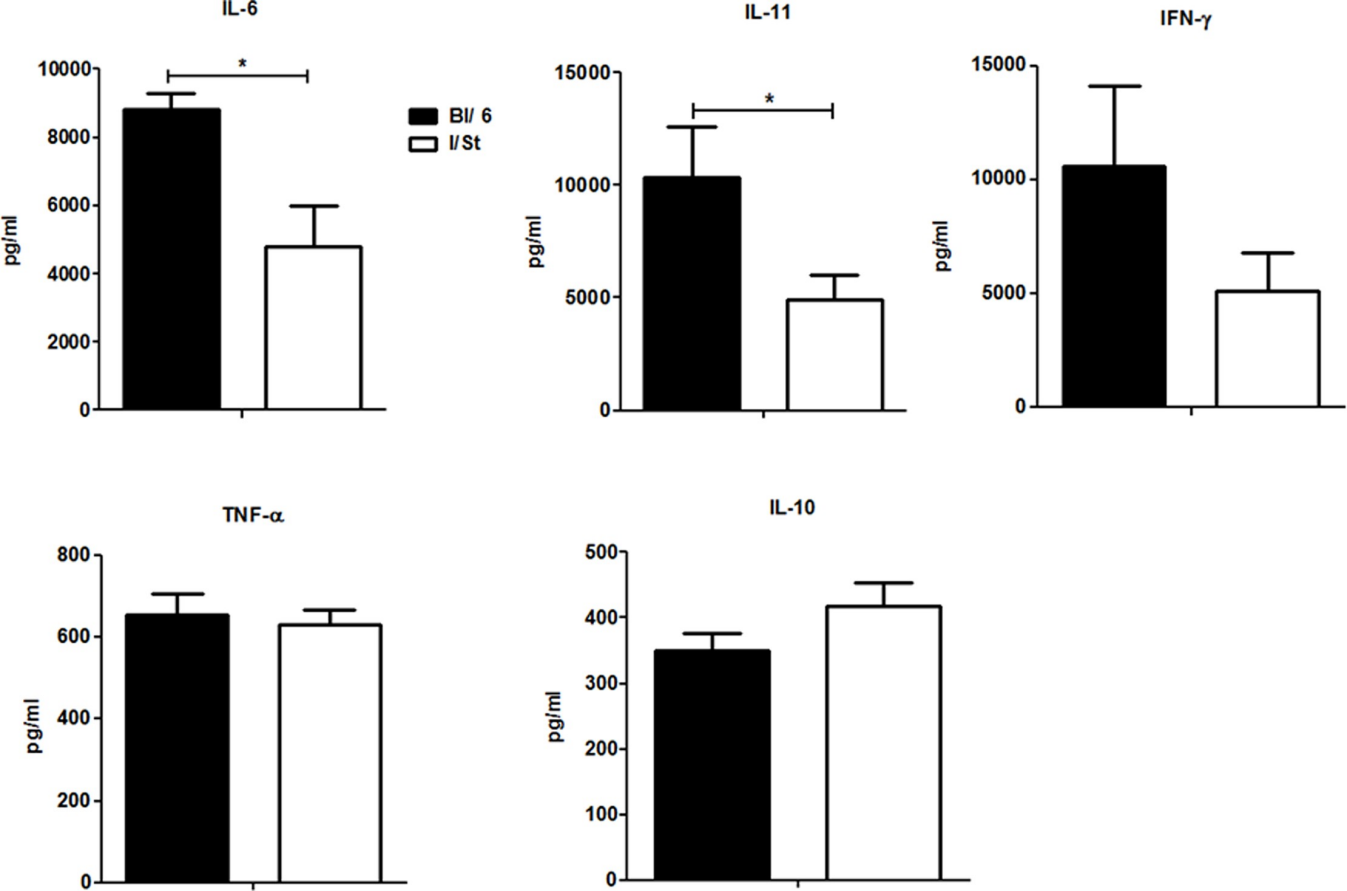
Cytokine production by cultured lung cells stimulated with mycobacterial antigens. Cytokine contents (IL-6, IL-11, IFN-γ, TNF-α and IL-10) in lung cell culture supernatants (see Materials and Methods) were assessed in dynamics in the ELISA format for 4 (B6) or 3 (I/St) mice per time point per group. The results are displayed as mean ± SEM. Asterisks * correspond to *P*< 0.05 (ANOVA). *P* = 0.13 for IFN-γ.

To find out what profiles of cytokine production underlined diverse pictures of lung pathology and inflammation in mice of the two strains, we assessed production of key regulatory and effector cytokines by lung cells at month 7 post infection. As shown in Fig 4, two major pro-inflammatory lung cytokines, IL-6 and IL-11, were secreted in significantly (*P* = 0.04 and *P* = 0.05, respectively, ANOVA) higher amounts by B6 lung cells, which corresponds well to the results obtained in the model with genetically attenuated mycobacteria [23]. Mice of the two strains did not differ by IL-10, TNF-α and, surprisingly, IFN-γ production. The latter result was unexpected, given that we constantly observed a higher IFN-γ response in B6 compared to I/St mice in different experimental settings [13, 23, 31]. However, the most likely explanation of this discrepancy is purely technical: only three I/St mice in appreciable health condition were available at this time point, which strongly increased statistical straggling, resulting in *P* = 0.057, i.e., borderline significance.

## 4. Discussion

Manifestations of *M*. *tuberculosis* infection in humans varies from an asymptomatic latent possession to a progressive fatal disease. Whereas the latter variant has been successfully reproduced in thousands of experimental studies and discussed in the context of drug and vaccine development (see, for example [32, 33]), adequate modelling of chronic, non-fatal, or even self-resolving, infection in rodents proved to be much more difficult [34, 35]. Meanwhile, only identification of essential immune mechanisms and biological markers of protection allow adequate modulation of the pathogenesis biochemical pathways and reliable assessment of novel vaccines and drugs performance [5]. To this end, we need affordable *in vivo* models.

Present work accomplishes a series of experimental studies characterizing pathology and lung immune responses during chronic and reactivation TB in B6 and I/St inbred mice with polar genetic susceptibility to infection [13, 14, 23, 26]. Taken together, these works clearly show that relying on substantial variability of host genetics and *M*. *tuberculosis* virulence it is possible to model a wide spectrum of human TB. In genetically susceptible mice, this includes reactivation infection in a short variant of a Cornell-like model, or a low-level asymptomatic mycobacterial possession after longer and more effective antibiotic treatment [13, 14]. In genetically resistant mice, we observed survival for more than 18 months after infection with attenuated *M*. *tuberculosis*, despite indefinite possession of mycobacteria and TB foci in the lungs ([24] and the present study).

In the extensive literature on chronic and latent TB (see [36] for review), it is emphasized that transition to latency, stability of infection control and sporadic reactivation equally depend upon genetics of the host and pathogen. More recently, the importance of double-edged genetic diversity in the human host–mycobacterial pathogen combination was non-ambiguously formulated by molecular epidemiologists [37, 38] and human geneticists [39], putting forward the role of host-pathogen coevolution. Nevertheless, combined studies on genetic variations within the two interacting species in a unified experimental system are rare, mostly due to a very long duration and high cost of such experiments. Our recent work [23] provided the grounds for the studying and manipulating the LTBI-like conditions in mice combining genetically attenuated *M*. *tuberculosis* with inbred mouse strains with polar genetic resistance. However, whilst characteristics of this model are suitable for evaluating the efficacy of potential drug and vaccine candidates against a small, persisting mycobacterial population at the late-phase of chronic infection, limited access to any unique genetically modified mycobacterial strain interferes with its wide application.

Results described above suggest that major parameters of infection induced by *Mtb*-acid strain are similar to those described for genetically attenuated strain. First, the difference in CFU counts between susceptible and resistant mice was highly significant throughout infection; in resistant animals, the numbers of mycobacteria in organs remained stable for 1.5 years (Fig 1), and no deaths were registered. Second, comparison of the results displayed in Figs 2–4 suggests that productive inflammatory immune responses supporting the existence of delineated TB foci in B6 lungs throughout infection provided effective protection. On the other hand, diffuse inflammation in I/St lungs, developing on the background of pro-inflammatory cytokine deficiency, triggered a very high level of T cell activation but ineffective protection. These observations are in full agreement with previous findings in the model based upon genetic attenuation of mycobacteria. We suggest that an extremely high content of activated T cells in genetically susceptible mice at month 7 post-challenge reflects an impaired influx of fresh T lymphocytes from circulation into diffusely inflamed lung tissue. Third, the pictures of pro-inflammatory cytokine production by lung cells (Fig 4) are similar to those obtained in the previous study [23].

Taken together, the results described above provide an argument for usage of similarly manipulated mycobacterial strains and mouse strains with different susceptibility to infection to assess new drug candidates in mice. Specifically, novel compounds aimed at combating two types of TB infection–fatal reactivation-like disease and chronic mycobacterial possession–may be studied in, respectively, genetically susceptible and resistant animals.
